# The influence of different helminth infection phenotypes on immune responses against HIV in co-infected adults in South Africa

**DOI:** 10.1186/1471-2334-11-273

**Published:** 2011-10-14

**Authors:** Zilungile L Mkhize-Kwitshana, Myra Taylor, Pieter Jooste, Musawenkosi LH Mabaso, Gerhard Walzl

**Affiliations:** 1 Offfice of the Deputy Dean: Postgraduate and Research, NRM School of Medicine, University of KwaZulu-Natal, P.O. Box 7, Congella, 4001, South Africa; 2 Department of Public Health Medicine, University of KwaZulu-Natal, P.O. Box 7. Congella, 4001 South Africa; 3 Nutritional Intervention Research Unit, P.O. Box 19070, Tygerberg, 7505, South Africa; 4 HIV/AIDS, STI and TB, Human Sciences Research Council, Private Bag X07, Dalbridge, Durban 4014, South Africa; 5 Department of Biomedical Sciences, University of Stellenbosch, Tygerberg, 7505, South Africa

## Abstract

**Background:**

The convergent distribution of the Human Immunodeficiency Virus (HIV) and helminth infections has led to the suggestion that infection with helminths exacerbates the HIV epidemic in developing countries. In South Africa, it is estimated that 57% of the population lives in poverty and carries the highest burden of both HIV and helmith infections, however, the disease interactions are under-researched.

**Methods:**

We employed both coproscopy and *Ascaris lumbricoides*-specific serum IgE to increase diagnostic sensitivity and to distinguish between different helminth infection phenotypes and their effects on immune responses in HIV co-infected individuals. Coproscopy was done by formol ether and Kato Katz methods. HIV positive and negative adults were stratified according to the presence or absence of *A. lumbricoides *and/or *Trichuris trichuria *eggs with or without elevated *Ascaris *IgE. Lymphocyte subsets were phenotyped by flow cytometry. Viral loads, serum total IgE and eosinophils were also analysed. Lymphocyte activation markers (CCR5, HLA-DR, CD25, CD38 and CD71) were determined. Non parametric statistics were used to describe differences in the variables between the subgroups.

**Results:**

Helminth prevalence ranged between 40%-60%. Four distinct subgroups of were identified, and this included egg positive/high *Ascaris*-specific IgE (egg^+^IgE^hi^), egg positive/low IgE (egg^+^IgE^lo^), egg negative/high IgE (egg^-^IgE^hi^) and egg negative/low IgE (egg^-^IgE^lo^) individuals. The egg^+^IgE^hi ^subgroup displayed lymphocytopenia, eosinophilia, (low CD4^+ ^counts in HIV^- ^group), high viral load (in HIV^+ ^group), and an activated lymphocyte profile. High *Ascaris *IgE subgroups (egg^+^IgE^hi ^and egg^-^IgE^hi^) had eosinophilia, highest viral loads, and lower CD4^+ ^counts in the HIV^- ^group). Egg excretion and low IgE (egg^+^IgE^lo^) status demonstrated a modified Th_2 _immune profile with a relatively competent response to HIV.

**Conclusions:**

People with both helminth egg excretion and high *Ascaris*-IgE levels had dysregulated immune cells, high viral loads with more immune activation. A modified Th_2 _helminth response in individuals with egg positive stools and low *Ascaris *IgE showed a better HIV related immune profile. Future research on helminth-HIV co-infection should include parasite-specific IgE measurements in addition to coproscopy to delineate the different response phenotypes. Helminth infection affects the immune response to HIV in some individuals with high IgE and egg excretion in stool.

## Background

The convergent distribution of the Human Immunodeficiency Virus (HIV) and helminth infections has been widely associated with the notion that persistent infection with helminths exacerbates the HIV epidemic in developing countries [1]. Chronic immune activation, altered immune cell distribution, immune suppression, altered cytokine profiles and strong T-helper 2 (Th_2_) bias induced by helminths, are suggested to increase susceptibility to the virus, enhancing its replication, increasing HIV disease severity and facilitating faster progression to AIDS [1,2]. The cellular and molecular immunological mechanisms of interaction reviewed in these papers [1,2], as well as many other epidemiological and immunological reports elsewhere and in Africa, provide sound suggestive evidence in support of the hypothesis [3-9].

South Africa (SA) has the highest number of HIV type 1 (HIV-1) infected individuals globally, about 5.6 million people out of a population of 48 million were living with HIV in 2010 [10]. Although the national estimates of helminth prevalence are not known, data from surveys in different SA provinces reveal infestation levels that range between 70-100% in school age children and preschoolers [11-17]. An estimated 57% of the SA population lives in poverty and carries most of the disease burden of the two infections [18,19]. However, studies that analyse the immunological interaction between these two disease conditions are limited in the country.

A major challenge in studies of co-infection with intestinal parasites is accurate laboratory diagnosis of the helminth infection, particularly in adults. In such studies, proper classification of helminth infection status is critical to avoid misinterpretation of results. It has been proposed that sole reliance on the presence of parasite eggs in stool to diagnose helminthiasis can lead to serious misinterpretation of results [20]. Maizels and Yazdanbakhsh [21] presented three phenotypic outcomes of helminth infection that are determined by antibody isotype (IgG_4 _and IgE) and T helper cell profiles. Each phenotype is characterised by specific immune responses to helminths. In the present study, stool egg detection has therefore been supplemented with serum *Ascaris lumbricoides *-specific IgE measurement. Four distinct subgroups, based on the presence or absence of stool eggs with or without elevated serum *Ascaris*-specific IgE were delineated. This paper reports the lymphocyte profiles including eosinophil counts, viral loads and the activation status in the defined subgroups.

## Methods

### Study design, setting and participants

Individuals in this study were a subgroup of adults (older than 18 years) from a larger prospective deworming study published in part elsewhere [20]. Ethical approval was obtained from the South African Medical Research Council and the University of Stellenbosch Ethics Committees. Permission to conduct the study was granted by the Matthew Goniwe Clinic management team. Written informed consent, which included permission to do HIV testing, was obtained from all participants.

The study was undertaken in Khayelitsha, Western Cape Province (SA), an informal settlement with limited resources, high helminth endemicity and HIV prevalence. A survey of 12 primary schools in this settlement showed that more than 90% of school children were infected by helminths [22], while a recall study on the history of helminth infection among adults showed that more than 70% had been infested by helminths previously [20]. Within the Western Cape Province, the prevalence of HIV in Khayelitsha (22%) was higher than the 9.1% provincial level [23].

**Figure 1:**
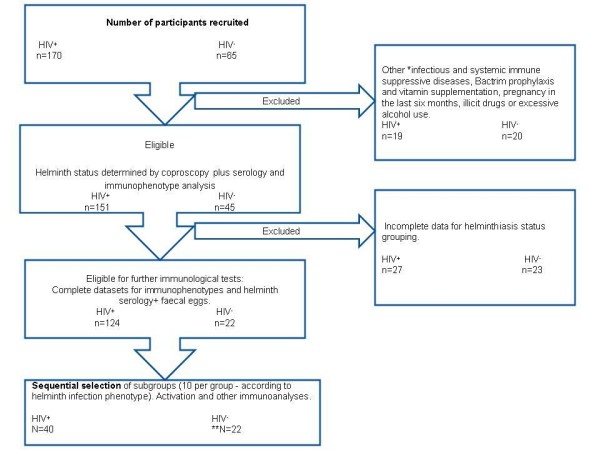
**Selection strategy for inclusion of participants for immunological analyses**. **Among the HIV^- ^eligible participants, 23 did not submit the complete set of samples thus the remaining 22 were included by default.

Study participants were recruited from Mathew Goniwe clinic between May 2002 and November 2003 for the main study [20] and the present analyses commenced in August 2004 to November 2005. The HIV positive individuals were purposively recruited from the HIV Positive Support Group at the clinic, while the HIV negative were adults accompanying patients to the clinic. The selection criteria for the present study are outlined in Figure 1. All participants were antiretroviral therapy naive as such treatment was not routinely available to communities in SA at the time. To exclude recent and present infectious diseases and possible recent treatment for worms, the participants and case record files were examined by the study clinician and one-to-one interviews undertaken. Two stool samples, collected on two consecutive days, and approximately 30 ml of blood were obtained from each participant. Females provided fresh urine samples for β-HCG pregnancy screening.

### Laboratory analyses

#### Detection of stool helminth eggs

Stool microscopy was performed by two independent microscopists using the formol-ether concentration [24] and the Kato Katz [25] methods respectively. Treatment with Mebendazole (and Praziquantel where indicated) was given to all participants with faecal helminth eggs. For this study, only participants infested with *A. lumbricoides *and/or *T. trichiura *were included.

#### HIV testing and viral loads

Confirmation of HIV status was done by a rapid test for HIV (InstantScreen^® ^Rapid HIV-1/2 Assay GAIFAR GmbH, Germany) at the clinic. Serum was re-tested at Tygerberg Academic Hospital, Department of Virology using the Abbott Axsym^® ^Microparticle Enzyme Immunoassay (MEIA) for the detection of antibodies to HIV-1 (subtypes M and O) and/or HIV-2. Confirmation of MEIA positive tests was done by PCR sequencing of viral DNA. HIV-1 viral load was determined by the Abbot LCx^® ^HIV RNA Quantitative assay (Abbott Q1Laboratories, IL) in the same Virology department.

#### Full blood counts and serum IgE tests

Serum total and *A. lumbricoides-*specific IgE levels were determined by CAP total IgE and ImmunoCAP^® ^Specific IgE (RAST), respectively. The cut-off for a positive Ascaris IgE test was > 0.35 ku/L, the assay detection limit. Full and differential blood counts were performed using the H2 Technicon Analyser at the Tygerberg Hospital Haematology laboratory on Ethylenediaminetetraacetic acid (EDTA) -anticoagulated whole blood. The full blood count results were used for the calculation of absolute lymphocyte subset numbers in the dual-platform analysis.

#### Four- Colour immunophenotyping of T cell subsets

Lymphocyte subsets were phenotyped by the MultiTest, four colour, direct immunofluorescence on the flourescence-activated cell sorter (FACS) Calibur™, Becton & Dickenson (BD) Biosciences, (San Jose, CA, USA). EDTA anticoagulated whole blood of 149 HIV-1 positive participants and 45 HIV-1 negative controls was stained with monoclonal antibodies (mAbs) to lymphocyte surface markers labelled with flourochromes as follows: Panel 1: CD45 Peridinin chlorophyll protein (PerCP); CD3 Flourescein Isothiocyanate (FITC); CD4 Allophycocyanin (APC); CD8 Phyco-erythrin (PE); Panel 2 CD3FITC;CD45 PerCP; CD19 APC; CD16+CD56 PE. All mAbs were obtained from Becton Dickinson (BD). Cells were stained within twenty four hours of blood collection using the standard procedure described in the BD MultiTest Reagent Package insert. Stained samples were analysed on the FACS Calibur™ (BD) Neon-Argon dual laser flow cytometer. For each sample, at least 10 000 events were acquired per tube using the MultiSET™ (BD) software. The percentages of each T and non-T lymphocyte subsets were determined. A double-platform method was then used to calculate the absolute counts of each lymphocyte subset from the total lymphocyte counts (from Haematology counts) and the lymphocyte percentages obtained by flow cytometry.

#### Peripheral blood mononuclear cell (PBMCs) preparation

PBMCs were separated by standard Ficoll Hypaque gradient centrifugation, suspended in fetal calf serum and 10% v/v Dimethylsulfoxide and cryopreserved in Liquid Nitrogen. Later, cells were thawed and counts were done by a phase-contrast microscope to simultaneously discriminate between viable and non-viable cells. Only samples with more than 90% viable cells were used.

#### Four-colour flow cytometric analysis of activation markers on PBMCs

Since the two chronic infections (HIV and helminths) result in sustained immune activation, hypothetically, upregulation of activation markers on peripheral lymphocytes would be more pronounced in co-infected individuals. To test this, the expression of CCR5, CD25, HLA-DR, CD38 and CD71 were determined on CD3^+^, CD4^+ ^and CD8^+ ^lymphocytes among HIV subgroups. These subgroups were randomly selected from all 124 HIV^+ ^eligible participants (every second participant, using laboratory identifier numbers until there were 10 participants per subgroup). All the 22 HIV negative, eligible participants with or without helminth infections were included by default, for comparisons by HIV status.

PBMCs from 40 HIV-1 positive participants and 22 HIV negative controls were labelled with directly conjugated monoclonal antibodies to various surface activation markers to determine the *ex-vivo *activation status. The procedure described in the BD Biosciences/Pharmingen Catalogue [26] was modified for microtiter plates. Each sample was stained in five different wells containing combinations of mAbs to surface markers directly conjugated to fluorochromes as follows: CD3- PerCP; CD4-APC; CD8-, HLA-DR- and CD71 -FITC; CD8-, CD38-, CD25- and CCR5-PE. The following isotype controls (BD) were included: IgG_1_k and IgG_2_a- FITC; IgG_1_k and IgG_2_a -PE; IgG_1_k -PerCP and IgG_2_a -APC. All mAbs conjugates were purchased from BD. Stained cells were analysed on the FACS Calibur™ flow cytometer using BD FACS™ tubes. A minimum of 10 000 events were acquired for each sample tube in list mode using the Cellquest software (BD). Lymphocytes were gated on established regions and percentages of CD3, CD4 and CD8 cells expressing CCR5, CD25, HLA-DR and CD71 were quantified.

#### Statistical analysis

Statistical analysis was conducted in STATA version 10.0 (Stata Corporation, College Station, Texas, USA). The normal distribution of recorded data was tested by the Shapiro Wilks tests and variables with skewed data were log-transformed. As some of the variables remained skewed after this transformation non-parametric tests were used. The median was used as a measure of central tendency for descriptive statistics. Kruskal Wallis was used to test for differences in the medians and for multiple comparisons of all measured variables between the subgroups in HIV positive and HIV negative groups. A. p-value ≤ 0.05 was considered to be statistically significant.

## Results

Of the 151 eligible, seropositive participants who submitted blood and stool samples, lymphocyte phenotypes were done on 149 individuals. Complete data for stratification of helminth infection was obtained for 124 individuals. Forty-five HIV negative participants submitted the samples and 39 were suitable for immunophenotypes and 22 could be stratified for helminthiasis. The demographic, viral and immunologic profile of the two HIV groups is summarized in Table 1. The reference ranges for the heamatological indices listed in this table had been established for the local population by the Tygerberg Hospital's Haematology Department. The local lower limits for CD4^+ ^and CD8^+ ^cells differ from those of the international reference ranges [27-29].

**Table 1:** Summary of baseline characteristics and haematologic indices of study participants by HIV status

Characteristics	HIV-1 Positive (n = 151)	HIV-1 Negative (n = 45)
**Demographic characteristics**		
Males	15	11
Females	136	34
Median Age (years)	30.6	40.0
**Serum IgE and Helminth Infection Status**		
Mean total IgE (Ref. range below 70 KU/L)	429.2 kU/L	655.18 kU/L
Mean *Ascaris*-specific IgE (Ref. range below 0.35 kU/L)	2.15 kU/L	2.86 kU/L
Helminth egg positive (number/total) %	(51/124)^a ^41.1	(18/39)^b ^46.1
Helminth egg positive and/or positive serology*	(99/151) 66.0	(32/45) 71. 1
Ascaris Worm Burden epg** Mean (SD)	170 (86.5)	191 (78.8)
Trichuris Worm Burden epg Mean (SD)	19.8 (36.7)	74.11 (56.2)
**Haematological/Viral Indices (Median)**	**(n = 149)**	**(n = 45)**
Total lymphocytes ( 1.0-4.0 × 10^9^/L)	1.98	2.22
T lymphocytes (1.1-1.7 × 10^9^/L )	1.49	1.71
CD8^+^(0.5-0.9 × 10^9^/L)	0.78	0.47
CD4^+ ^(0.7-1.1 × 10^9^/L)	0.32	0.74
CD4:CD8 ratio 1-1,5	0.4	1.58
NK cells (0.2-0.4 × 10^9^/L)	0.1	0.18
B lymphocytes(0.2-0.4 × 10^9^/L)	0.1	0.24
Median Viral load (copies/ml)	33 317	-

*Evidence of helminth exposure as indicated by faecal egg excretion and/or elevated *Ascaris lumbricoides *specific IgE. ** Epg is eggs per gram of stool. ^a ^Twenty seven of 151 (HIV^+^) and^b ^six of 45 ( HIV^-^) participants did not submit faecal samples. The haematological reference values in brackets were established for the local population at Tygerberg Hospital and their lower limits differ from those of the CDC reference ranges for CD4^+ ^(0.475-1.616 × 10^9^/L) and (CD8^+^:0.209- 0.924 × 10^9^/L) [27-29].

Serum total IgE levels were 6-fold above the method reference range among the HIV-1 sero-positive people and 9-fold higher in the HIV-1 uninfected group while *Ascaris*-specific IgE was 6 times and 8 times higher in the two groups, respectively. The proportions by combined measures of high serum *Ascaris*-specific IgE plus stool egg positivity as indicators for helminth infection (>65%) exceeded the percentage of participants with stool egg positivity only (>40%) in both HIV positive and negative groups (Table 1). A range of parasites was detected in this adult population, including infection with more than one species. The most prevalent infections were *A. lumbricoides (*44 of the 51 HIV^+ ^egg positive and 14 of 18 HIV^- ^egg positive participants), followed by *T. trichiura (*30 of 51 HIV^+ ^and 8 of 18 HIV^- ^participants). Other parasites included the *Taenia *species (5 of 51 and 3 of 18 HIV^+ ^and HIV^- ^individuals respectively), *Fasciola *(one in each of the two HIV-1 groups) and *Schistosoma mansoni (*one of the 18 HIV-1 negatives). Among the HIV-1^+^, 21 of 51 (41%) were dually infected by *A. lumbricoides *and *T. trichiura *and 5 out of 18 (28%) among HIV^- ^group harboured both these parasites. Worm burdens were lower in the HIV positive group.

### Stratification of helminth infection subgroups by coproscopy and *A. lumbricoides *specific IgE (*Ascaris *IgE) serology

Some participants excreted parasite eggs with or without elevated *A. lumbricoides *IgE while others had high serum *A. lumbricoides *IgE but did not excrete eggs. Four distinct subgroups were stratified, among the HIV positive individuals. Subgroup 1 (n = 21) comprised of *Trichuris *and/or *Ascaris *egg positive stool and elevated *Ascaris *IgE (egg^+^IgE^hi^), a typical infection, subgroup 2 (n = 35) consisted of *Trichuris *and/or *Ascaris *egg positive stool without elevated *Ascaris *IgE (egg^+^IgE^lo^), subgroup 3 (n = 21) contained *Trichuris *and *Ascaris *egg negative stool but elevated *Ascaris *IgE (egg^-^IgE^hi^), and subgroup 4 (n = 47) included *Trichuris *and *Ascaris *egg negative stool and low *Ascaris *IgE (egg^-^IgE^lo^). Among the HIV negative participants the subgroups were egg^+^IgE^hi ^(n = 9) egg^+^IgE^lo ^(n = 9), egg^-^IgE^hi ^(n = 11) and egg^-^IgE^lo ^(n = 10).

### Lymphocyte subsets, eosinophils and viral load in subgroups

Total lymphocyte numbers and their subpopulations (T, B, NK, CD4^+ ^and CD8^+^), eosinophil counts and viral load were assessed in the four subgroups in HIV infected and uninfected participants (Tables 2 and 3).

**Table 2:** Comparison of eosinophils, viral load and lymphocytes among HIV positive subgroups

SUBGROUPS					
**Cell Types^†^**	**Egg^+^IgE^hi ^(n = 21)**	**Egg^+^IgE^lo ^(n = 35)**	**Egg^-^IgE^hi ^(n = 21)**	**Egg^-^IgE^lo ^(n = 47)**	***p-value***

Eosinophils	0.32	0.15	0.18	0.10	0.01
Total lymphocytes	1.72	2.1	1.89	1.98	0.50
T lymphocytes	1.29	1.75	1.39	1.25	0.23
CD8+	0.66	0.84	0.78	0.66	0.66
CD4+	0.28	0.41	0.32	0.23	0.01
CD4:CD8 ratio	0.38	0.48	0.37a	0.34	0.03
NK lymphocytes	0.11	0.12	0.10	0.11	0.83
B lymphocytes	0.09	0.15	0.12	0.10	0.03
Viral Load	70 878	25 666	87 813	28 257	0.12

^†^Cells are expressed medians in count × 10^9^/L and viral load in copies per ml of blood.

**Table 3:** Comparison of eosinophils and lymphocytes among HIV negative subgroups

SUBGROUPS					
**Cell Types^†^**	**Egg^+^/IgE^hi ^(n = 9)**	**Egg^+^/IgE^lo ^(n = 9)**	**Egg^-^/IgE^hi ^(n = 11)**	**Egg^-^/IgE^lo ^(n = 10)**	***p-value***

Eosinophils	0.37	0.12	0.31	0.22	0.09
Total lymphocytes	1.95	2.79	2.65	2.37	0.05
T lymphocytes	1.40	1.91	1.73	1.83	0.14
CD8+	0.36	0.49	0.47	0.41	0.49
CD4+	0.67	0.75	0.68	0.76	0.59
CD4:CD8 ratio	1.86	1.54	1.37	1.57	0.47
B lymphocytes	0.08	0.17	0.23	0.15	0.01
NK lymphocytes	0.22	0.28	0.25	0.24	0.16

^†^Cells are expressed as medians in count × 10^9^/L blood.

### HIV singly-infected (egg^-^lgE^lo^) versus the HIV and typical helminth co-infected (egg^+^IgE^hi^) subgroup

The HIV-1^+^, egg^-^IgE^lo ^subgroup had the lowest CD4^+ ^cells, T-lymphocyte counts and CD4:CD8 ratio than all the subgroups. In addition, there was no statistically significant difference between HIV^+^egg^+^IgE^hi ^and the HIV^+^egg^-^IgE^lo ^subgroups in all measured variables except for eosinophils which were significantly higher in the dually infected individuals (Table 2).

### HIV negative helminth uninfected (egg^-^IgE^lo^) versus typical helminth infected (egg^+^IgE^hi^) subgroup

Higher but not statistically significant absolute counts for all T and non-T lymphocyte subsets were observed when the egg^-^gE^lo ^was compared to the typical helminth infected subgroup (egg^+^IgE^hi^). The egg^-^gE^lo ^subgroup had both marginally non significant higher total lymphocyte counts (p = 0.06) and lower eosinophils (p = 0.08). However, the CD4:CD8 ratio was higher in the egg^+^IgE^hi ^subgroup.

### Typical helminth (egg^+^IgE^hi^) and HIV co-infected subgroup

The HIV positive, egg^+^IgE^hi ^subgroup had lymphocytopenia and statistically significant eosinophilia. Amongst the subgroups with evidence of dual infection, in the HIV positive egg^+^IgE^hi ^group, the absolute values were lower for all lymphocyte populations, compared to the overall absolute counts for the entire HIV positive group (Table 1). The absolute values for all lymphocyte populations in the HIV positive egg^+^IgE^hi ^subgroup were also lower compared to the egg^+^IgE^lo ^and the egg^-^IgE^hi ^subgroups (Table 2).

B lymphocytes were significantly lower in the egg^+^IgE^hi ^subgroup (p = 0.03) and CD4^+ ^counts were marginally non-significantly lower (p = 0.06) compared to the egg^+^IgE^lo ^subgroup.

The egg^+^IgE^hi ^status was associated with more frequent eosinophilia compared to the other three subgroups and this difference was highly significant (p = 0.01) between the egg^+^IgE^hi ^subgroup and the egg^-^IgE^lo ^subgroup (no evidence of worm exposure) (Table 2). Furthermore, the median viral load for the egg^+^IgE^hi ^subgroup was more than 2-fold higher than that of the combined HIV^+ ^group shown in Table 1, and nearly 3-fold higher than those of the egg^+^IgE^lo ^and egg^-^IgE^lo ^subgroups (Table 2). These were however not statistically significant (p = 0.12).

### HIV negative egg^+^IgE^hi ^subgroup

The HIV negative, egg^+^IgE^hi ^subgroup had lymphocytopenia and eosinophilia. As in the HIV positives, the HIV uninfected, egg^+^IgE^hi ^participants had lower median values for all lymphocyte subsets except the CD4:CD8 ratio that was highest (Table 3). Total and B lymphocytes were significantly lower in the egg^+^IgE^hi ^compared to the egg^-^IgE^hi ^subgroup (p = 0.05). Furthermore, the egg^+^IgE^hi ^subgroup had marked eosinophilia compared to the rest of the HIV-1 negative subgroups and the absolute eosinophil counts were higher in this egg^+^IgE^hi ^group compared to both subgroups with low IgE(egg^+^IgE^lo^) and the (egg^-^IgE^lo^).

### HIV positive Egg^+^IgE^lo ^subgroup

In the HIV positive group, the egg^+^IgE^lo ^participants had highest absolute numbers for all the lymphocyte subsets than all the other subgroups (Table 2), and also higher than the overall values for the entire HIV positive group shown in Table 1. There were significant differences in CD4^+ ^(p = 0.01) and CD4:CD8 ratio (p = 0.03) between the egg^+^IgE^lo ^and the egg^-^IgE^lo ^subgroups. B lymphocyte values were significantly higher in the egg^+^IgE^lo ^compared to the egg^+^IgE^hi ^subgroup (p = 0.02). Additionally, the egg^+^IgE^lo ^subgroup had the lowest viral load although this was not statistically significant (p = 0.12). However, a partially significant difference in viral load was observed between egg^+^IgE^lo ^and the egg^-^IgE^hi ^subgroup (p = 0.05) when two extreme outliers are excluded from the analysis (Table 2).

### HIV negative Egg^+ ^IgE^lo ^subgroup

Similarly, in the HIV uninfected group, egg^+^IgE^lo ^participants appeared to have the highest total-, T-, CD8^+-^- and NK-lymphocyte counts. The total lymphocytes were significantly higher in this subgroup compared to the egg^+^IgE^hi ^subgroup. The CD4^+ ^and CD4:CD8 ratio was similar to the values for the egg^-^IgE^lo ^subgroup (without evidence of helminth infection) (Table 3).

### HIV positive egg^+^IgE^hi ^and egg^-^IgE^hi ^(high IgE) subgroups

Among the HIV infected participants, both subgroups with elevated IgE (egg^+^IgE^hi ^and egg^-^IgE^hi^) had the highest viral loads with 70 878 copies per ml (cpml) and 87 813 cpml respectively compared to the two subgroups with low *Ascaris *IgE egg^+^IgE^lo ^and egg^-^IgE^lo ^which had 25 666 cpml and 28 257 cpml, respectively. Although these differences in viral load were not statistically significant, the two subgroups with high IgE had almost three-fold higher viral loads. In addition to higher virus burden, the egg^+^IgE^hi ^subgroup also had relatively higher eosinophil levels (Table 2).

### HIV negative egg^+^IgE^hi ^and egg^-^IgE^hi ^subgroups

Likewise, among the HIV uninfected individuals, both groups with high IgE (egg^+^IgE^hi ^and egg^-^IgE^hi^) had marked eosinophilia and lower CD4^+ ^counts. Other observations in this section included the findings that low IgE (egg^+^IgE^lo ^and egg^-^IgE^lo^) subgroups also had low eosinophil counts, particularly in the HIV uninfected groups (Table 3).

### Viral loads and levels of immunodeficiency in subgroups

**Figure 2:**
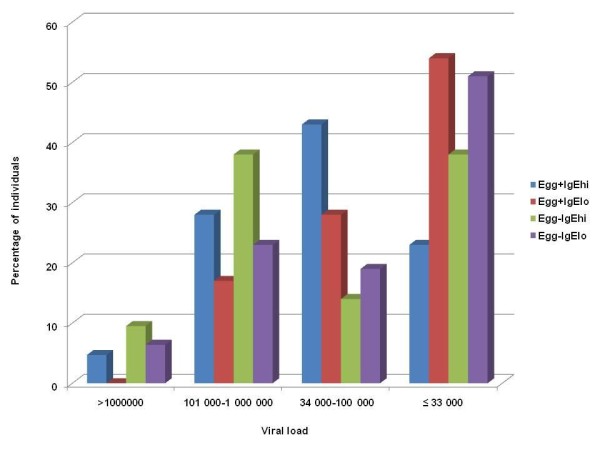
**Levels of viral loads (copies per ml of blood) in the four subgroups of HIV infected participants**.

**Figure 3:**
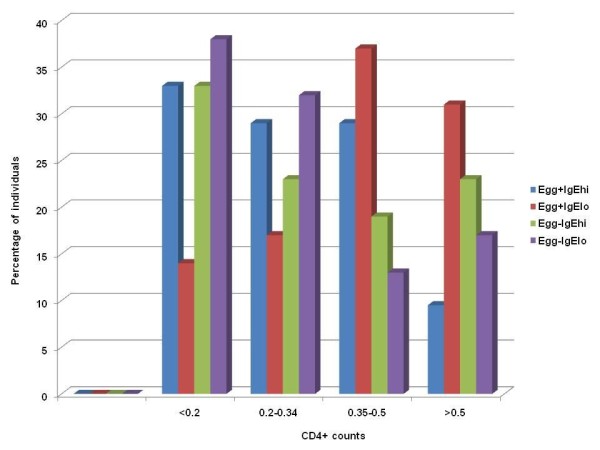
**Levels of immunodeficiency by CD4 counts (cells per ml of blood) in the four subgroups of HIV infected participants**.

The maximum viral loads in each subgroup were 1 013 265 cpml (egg^+^IgE^hi^); 448 447 cpml (egg^+^IgE^lo^); 1 711 249 cpm (egg^-^IgE^hi^) and 3 637 277 cpml (egg^-^IgE^lo^). In the latter, three individuals had virus burden exceeding 1 000 000 cpml, and the individual with the highest viral load in the entire HIV group was found in this subgroup. The viraemia, determined by different grades of virus burden from 33 000 cpml (based on the median for all HIV positive study individuals) (Figure 2), and levels of immune deficiency, determined by CD4^+ ^counts (Figure 3) were assessed in the four subgroups. The egg^+^IgE^hi ^subgroup had the lowest number of individuals with high CD4^+ ^counts and lowest proportion with low viral load. The egg^+^IgE^lo ^group had the highest number of individuals with higher CD4^+ ^counts and lower viral loads (Figure 2 and Figure 3).

### Immune Activation profile in HIV positive and HIV negative groups

#### HIV positive subgroups

**Figure 4:**
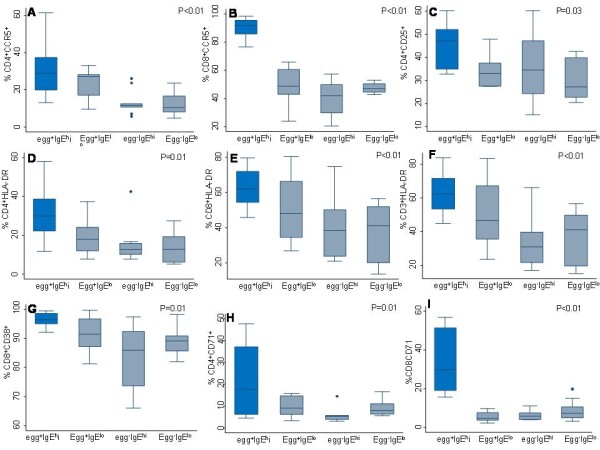
**Box and whisker plots for lymphocyte surface expression of activation markers in HIV^+ ^subgroups, and Kruskal Wallis Anova comparison of medians for significant differences between subgroups (at p value ≤ 0.05), where Egg^+^IgE^hi ^denotes helminth egg positive and elevated *Ascaris lumbricoides *IgE subgroup, Egg^+^IgE^lo ^represents egg positive and low *A***. *lumbricoides *IgE subgroup, Egg^-^IgE^hi ^designates egg negative and high *A. lumbricoides *IgE subgroup, and Egg^-^Ig^lo ^stands for helminth egg negative and low *A. lumbricoides *IgE subgroup.

Figure 4 illustrates interactions between the four subgroups of the 40 selected HIV positive participants. The egg^+^IgE^hi ^subgroup had a statistically significant increased expression of all activation markers. When the HIV and helminth co-infected, egg^+^IgE^hi ^subgroup was compared to the HIV-singly infected egg^-^IgE^lo ^subgroup, both had similar median CD4^+ ^counts, which were slightly higher with 0.27 × 10^9^/L cells in the former compared to 0.22 × 10^9^/L cells in the egg^-^IgE^lo ^subgroup. The median viral load was significantly higher in the dually infected egg^+^IgE^hi ^subgroup with 101 007 cpm compared to 4 234 cpm in the egg^-^IgE^lo ^subgroup (p = 0.01).

It is noted that the viral loads and CD4^+ ^counts could not be matched before random selection of the subgroups. Comparisons of activation between these two subgroups showed that expression of all activation markers was almost two-fold higher in the egg^+^IgE^hi ^subgroup. These results showed a significant difference between the egg^+^IgE^hi ^helminth/HIV co-infected subgroup and the HIV-singly infected helminth non-infected (egg^-^IgE^lo^) subgroup with regards to the level of immune activation.

Further analysis showed that the egg^+^IgE^hi ^helminth/HIV co-infected subgroup expressed significantly higher levels of all activation markers than all the other subgroups in the HIV positive group (Figure 4). All these differences were statistically highly significant (p≤ 0.01) in all variables (Figure 4) except for CD4^+^CD25^+ ^(p = 0.03). In this subgroup, median CD3^+^, CD4^+ ^and CD8^+ ^expression of the classic activation marker, HLA-DR, was higher than in all the other three subgroups (Figure 4 panels D-F, p≤ 0.01). In addition, median expression of CCR5 by CD8^+ ^cells exceeded 90% while in CD4^+ ^cells median CCR5 expression was almost twice as high as in the egg^-^IgE^hi ^and the egg^-^IgE^lo ^subgroups.

Furthermore, the early activation marker-CD71 was 2-3 fold higher (p < 0.001) than in the other three subgroups in both the CD4^+ ^and CD8^+ ^compartments (Figure 4 Panels H and I). Almost all the CD8^+ ^cells were CD38^+ ^in this subgroup and this was statistically significantly higher (p = 0.01) than in the other subgroups (Figure 4, Panel G).

The highest levels of percentages of lymphocytes expressing all activation markers by the egg^+^IgE^+ ^subgroup is remarkable in view of the fact that in the preceding section this egg^+^IgE^hi ^group was shown to have a tendency towards a generalized reduction of lymphocyte populations.

In addition, stool egg positive subgroups (egg^+^IgE^hi ^and egg^+^IgE^lo^) had more than two-fold median CCR5 expression by CD4^+ ^cells, compared to the stool egg negative subgroups (egg^-^IgE^hi ^and egg^-^IgE^lo^) in the HIV^+ ^group. Furthermore, in both stool egg positive subgroups, median expression of CD38 by CD8^+ ^lymphocytes exceeded 90% and this was statistically significant (Figure 4).

#### HIV negative subgroups

In the HIV^- ^negative group, the egg^+^IgE^hi ^subgroup had lower CD4+ counts compared to the other three subgroups. No dramatic increases in activation markers were observed among these individuals and no statistically significant differences were observed in any of the variables analysed except for the apparent increase (nearly two-fold) in median CD4^+ ^CCR5^+ ^in the egg^+^IgE^hi ^and egg^+^IgE^lo ^subgroups (4.3% and 4.9%) compared to the egg^-^IgE^hi ^and the egg^-^IgE^lo ^subgroups (2.2% and 2.4%) respectively. Likewise, expression of CCR5 on CD8^+ ^cells was higher in the egg^+^IgE^hi ^compared to the egg^-^IgE^hi ^subgroup (p = 0.09). A marginally non-significantly higher CD8^+ ^HLA-DR was also observed in the egg^+^IgE^lo ^compared to the egg^-^IgE^hi ^subgroups (p = 0.09).

## Discussion

In this study the combined use of coproscopy and serology not only improved the diagnosis of helminthiasis, but also facilitated the distinction of four phenotypically different infection profiles: (i) stool egg positive and high IgE (egg^+^IgE^hi^), (ii) the stool egg positive and low IgE (egg^+^IgE^lo^), (iii) the stool egg negative and high IgE (egg^-^IgE^hi^) and (iv) the egg negative, IgE low (egg^-^IgE^lo^) subgroups. Similarly, Maizels and Yazdanbakhsh [21] also described three phenotypic outcomes of helminth infections. In the present study, different forms of immune alterations and their possible effect on HIV infection were assessed within the defined subgroups.

Overall the prevalence of intestinal helminth infections was high (40-60%) in adults residing in this resource-limited study setting. Furthermore, the participants presented with a high IgE responder profile as shown by more than six-fold total and specific IgE above the reference ranges in both HIV^+ ^and HIV^- ^groups. This finding concurs with earlier suggestions that Africans generally present with elevated IgE levels as demonstrated in studies conducted in a similar ethnic group [30,31]. IgE class switching is mediated by CD4^+ ^Th_2 _cells [32], and it is at present unclear whether the high IgE in this population is due to a genetic predisposition or environmental influences that mediate Th_2 _cell predominance.

The expected hypothetical study outcome was that dual infection would adversely impact on the immune profile of affected hosts compared to singly-or non-infected counterparts. Comparisons between singly and dually-infected HIV positive subgroups revealed no significant differences in lymphocyte profiles. There was significant eosinophilia in the HIV-helminth co-infected subgroup. In the absence of HIV infection, a tendency to increased lymphocytes and marginally lower eosinophils was observed in the egg^-^IgE^lo ^compared to the typical helminth infected egg^+^IgE^hi ^subgroup. The differences in the other cell types were not statistically significant between these two groups. The relatively smaller numbers in the HIV negative subgroups could have influenced the power to obtain statistically significant differences. The fact that no such differences were noted in the HIV positive subgroups suggests that HIV-induced immunosuppression could be responsible for masking any differences in the latter. Firstly the HIV^+^, egg^-^IgE^lo ^subgroup had the lowest median CD4^+ ^counts. Secondly, similar percentages of participants in the egg^+^IgE^hi ^and egg^-^IgE^lo ^subgroups were severely immunocompromised (less than 0, 2 × 10^9 ^cells/L CD4^+ ^counts) and virus burden was similar in the two subgroups (Figure 3).

When the dually-infected subgroups were analysed, several observations revealed that certain immunological phenoytypes of helminth infection may favour HIV replication, thus by inference lending support to the study hypothesis that helminthiasis might enhance virus replication. Firstly, typical helminth infection (as reflected by the egg^+^IgE^hi ^status) was accompanied by eosinophilia, approximately three-fold higher viral loads and generally lower absolute counts for all lymphocyte populations when compared to the other three subgroups. This tendency was observed in both HIV positive and negative groups. This finding concurs with the report that chronic helminth infections in adults resulted in disruptions in peripheral T cell populations [33]. In addition, all measured activation markers were significantly elevated in the egg^+^IgE^hi ^and HIV co-infected subgroup. Nearly all the CD8^+ ^cells were CD38-positive in the egg^+^IgE^hi ^subgroup (Figure 4). Immune activation has been widely implicated as playing a pivotal role in HIV pathogenesis through various pathways [1,2,34-36].

The observed decrease in lymphocyte populations among the egg^+^IgE^hi ^individuals in this study, could indirectly relate to a compromised immunological ability to respond to HIV infection. Lymphocytes play a pivotal role in immune response to infection in general and in containing the HI virus [32]. Eosinophils are proposed to increase the number of activated cells that are infectable with HIV since they express the CD4 receptor molecule and *in vitro *studies showed that when these cells are activated, they can be infected by HIV [37,38]. These suggestions are in agreement with the study hypothesis and findings.

Furthermore, both subgroups with elevated IgE (egg^+^IgE^hi ^and egg^-^IgE^hi ^had eosinophilia, low CD4^+ ^counts (especially in the HIV^- ^group) and three-fold higher viral load (in HIV^+^group) compared to the low IgE subgroups (the egg^+^IgE^lo ^and egg^-^IgE^lo^). Both eosinophilia and high IgE are classic Th_2 _responses that are universally induced by helminth infections [21,32]. The association of these mediators with higher viral loads supports the concept that Th_2 _polarisation by helminths suppresses the protective Th_1 _responses and hence promotes HIV replication [1,39,40].

In direct contrast to these responses of high IgE subgroups, the egg^+^IgE^lo ^subgroup had significantly higher absolute CD4^+ ^counts and helper/suppressor ratios and generally higher absolute numbers of all lymphocyte subsets accompanied by the lowest viral load. This finding suggests a low IgE phenotype with a better ability to control the HIV viral infection in these individuals presenting as modified Th_2 _responders [21].

Several limitations are noted in this study. The cross sectional design was a major shortcoming as both HIV and helminth infections are chronic conditions. HIV infection has different stages, each characterised by different pathogenesis and immunologic features. Likewise, helminth infections have different life cycle phases that are associated with specific immune responses. Thus, an ideal design for studies of co-infections with these two organisms would be a prospective cohort study with randomised sampling. The small sample size also limited the study. The majority of participants were females in both HIV positive and negative groups and the HIV negative participants were slightly older (10 years difference in median ages) than the HIV positive group. Both age and gender may affect many immunological and haematological parameters [1]. These factors could possibly confound the study; nevertheless, it was encouraging to find some significant results despite the listed limitations.

## Conclusions

Our results suggest that HIV immune responses are impaired by helminth infections in certain susceptible groups of individuals, particularly in individuals who excrete worm eggs and have high parasite IgE in serum. Helminth-induced Th_2 _bias is also associated with impaired immune response to HIV. Individuals with a modified Th_2 _response to helminths are better able to control HIV.

The present work contributes to the body of new knowledge in South Africa and provide evidence that the presence of intestinal parasite eggs in stools of infested individuals represents only a part of the helminth infection phenotype, which can be further delineated by levels of helminth-specific IgE (and IgG_4_) [21]. Grouping according to stool egg positivity alone would misclassify infection and non-infection; obscure the recognition of additional phenotypes with major implications for the interpretation of studies addressing immunological effects of co-infections with helminths. This has implications for the design of future studies aimed at analysing helminth co-infection and more importantly, interpretation of such studies. Parasite IgE serology should be used to supplement egg detection and delineate the different response phenotypes in future co-infection studies.

## Competing interests

The authors declare that they have no competing interests.

## Authors' contributions

ZLM-K: Involved during conceptualisation of the project, questionnaire design and field-testing, undertaking all immunological laboratory tests, collecting all data, analysing the results, intellectual input and writing the manuscript.MT: Co-supervisor for the project, Intellectual input, writing and critical editing of the manuscript. PJ: Critical reading of the manuscript. MLHM: Writing and critical editing of the manuscript. GW: Conceptualization of the project, intellectual input and supervision of the whole project and all the immunological work, writing of the manuscript. All authors read and approved the final manuscript.

## Pre-publication history

The pre-publication history for this paper can be accessed here:

http://www.biomedcentral.com/1471-2334/11/273/prepub
